# Conjugation across *Bacillus cereus* and kin: A review

**DOI:** 10.3389/fmicb.2022.1034440

**Published:** 2022-11-04

**Authors:** Pauline Hinnekens, Nancy Fayad, Annika Gillis, Jacques Mahillon

**Affiliations:** ^1^Laboratory of Food and Environmental Microbiology, Earth and Life Institute, Louvain-la-Neuve, Belgium; ^2^Multi-Omics Laboratory, School of Pharmacy, Lebanese American University, Byblos, Lebanon

**Keywords:** *Bacillus anthracis*, *Bacillus cereus*, conjugation, plasmid, T4SS, *Bacillus thuringiensis*, *Bacillus mycoides*, *Bacillus cytotoxicus*

## Abstract

Horizontal gene transfer (HGT) is a major driving force in shaping bacterial communities. Key elements responsible for HGT are conjugation-like events and transmissible plasmids. Conjugative plasmids can promote their own transfer as well as that of co-resident plasmids. *Bacillus cereus* and relatives harbor a plethora of plasmids, including conjugative plasmids, which are at the heart of the group species differentiation and specification. Since the first report of a conjugation-like event between strains of *B. cereus sensu lato* (*s.l.*) 40 years ago, many have studied the potential of plasmid transfer across the group, especially for plasmids encoding major toxins. Over the years, more than 20 plasmids from *B. cereus* isolates have been reported as conjugative. However, with the increasing number of genomic data available, *in silico* analyses indicate that more plasmids from *B. cereus s.l.* genomes present self-transfer potential. *B. cereus s.l.* bacteria occupy diverse environmental niches, which were mimicked in laboratory conditions to study conjugation-related mechanisms. Laboratory mating conditions remain nonetheless simplistic compared to the complex interactions occurring in natural environments. Given the health, economic and ecological importance of strains of *B. cereus s.l.*, it is of prime importance to consider the impact of conjugation within this bacterial group.

## Introduction

Genetic diversity is key for long-term survival of various organisms, be it micro-ones like bacteria or fungi, all the way to plants and animals, in continuously changing ecosystems. For bacteria, although their reproduction is mostly asexual by binary fission, they have developed the capacity to diversify their genetic content and to acquire DNA material from relatively distant organisms, by horizontal gene transfer (HGT; Arnold et al., 2021). Defined as “the non-genealogical transmission of genetic material from one organism to another” (Goldenfeld and Woese, 2007), HGT has a strong impact on bacterial genomes by increasing the potential of acquiring advantageous genes, resulting in better adaptation and colonization of new ecological niches (Soucy et al., 2015; McInerney et al., 2017). Diverse mechanisms drive HGT among prokaryotes, and the three major mechanisms are natural transformation, transduction and conjugation (von Wintersdorff et al., 2016). While transformation consists in the capture of foreign DNA from the environment (Mell and Redfield, 2014) and transduction is mediated by bacteriophages (a.k.a. phages; Colavecchio et al., 2017), conjugation requires a physical contact between the cell partners prior to transfer of plasmid DNA (de la Cruz et al., 2010). Aside from the three major mechanisms of HGT, several non-canonical mechanisms of DNA transfer have been identified, including the so-called “gene-transfer agents,” transfer *via* membrane vesicles and intercellular nano-tubes (Arnold et al., 2021). Yet, these non-canonical modes of HGT are beyond the scope of this review.

The mechanism of conjugation – or conjugative transfer – occurs in a stepwise process and requires a secretion system, consisting of a type IV secretion system (T4SS), and a replication machinery to obtain two copies of the conjugative element at the end of the transfer event (for detailed reviews on conjugation mechanism, refer to Cabezón et al., 2015 and Virolle et al., 2020). During this process, a relaxase first recognizes the origin of transfer (*oriT*) of the conjugative element, nicks the *oriT* in a strand-specific manner and remains bound to the nicked strand. The resulting nucleoprotein complex is then recruited to the T4SS by a coupling protein (CP) and replication of the intact strand takes place in the donor cell, displacing the nicked strand for transfer and further replication in the recipient cell (de la Cruz et al., 2010). T4SS are usually made of three ATPases, including one CP and two motor ATPases, one peptidoglycan hydrolase and several membrane proteins inserted into the donor cell envelop and forming the core of the T4SS complex (for detailed description of T4SS, refer to Ilangovan et al., 2015 and Waksman, 2019). Studies have mainly focused on the conjugative systems of Gram-negative bacteria, but the global mechanism of conjugative transfer is conserved among Gram-positive bacteria, despite structural differences in the T4S machinery (for a review on Gram-positive conjugation systems, refer to Goessweiner-Mohr et al., 2013).

Conjugative plasmids are capable of self-transfer and encode all the genetic material necessary for a functional conjugative apparatus. Some plasmids, called mobilizable plasmids, lack the genes encoding for the T4SS, but usually encode an *oriT* and a relaxase. They can hijack the transfer apparatus of a co-resident conjugative plasmid to trigger their transfer (Fernández-López et al., 2014). Suh transfer process is called mobilization. Other plasmids remain however non-transmissible and are often called non-mobilizable, as they lack all the elements necessary for conjugation-like movements, i.e., an *oriT*, a relaxase and a T4SS (Smillie et al., 2010).

As suggested by its large diversity of plasmids and mobile genetic elements (MGEs), the *B. cereus* group is of prime interest when it comes to conjugation and conjugation-related plasmid transfer. The *B. cereus* group, or *B. cereus sensu lato* (*s.l.*), refers to a group of closely related bacterial species, yet displaying a large ecological diversity. *B. cereus* group members are ubiquitous sporulating Gram-positive bacteria, mainly studied because several members of the group are of health, environmental or commercial interests or concerns (Ramarao et al., 2015). Within this review, we look back at the earlier work as well as at the data collected on *B. cereus* conjugation and conjugative plasmids described so far. We also examine the proportion of potential conjugative plasmids among the *B. cereus s.l.* genome sequences available to date. Overall, we aim at providing a full picture on conjugative plasmids residing within the plasmid-rich group of *B. cereus* and their implications in both laboratory and environmental-like conditions, as known to date.

## *Bacillus cereus s.l.*, plasmid-borne toxins and horizontal gene transfer

Three main species form the core of the *B. cereus* group: the opportunistic pathogen *B. cereus sensu stricto* (*s.s.*), potentially associated with foodborne infections, the etiological agent of anthrax, *B. anthracis*, and the entomopathogen *B. thuringiensis*, well-known for its biopesticide application (Kolstø et al., 2009; Ehling-Schulz et al., 2019). In addition, the *B. cereus* group includes other species, like the rhizoid-growing *B. mycoides* and *B. pseudomycoides* (Flügge, 1886; Nakamura, 1998), the psychrotrophic *B. weihenstephanensis* (Lechner et al., 1998; Hoton et al., 2009) – recently reclassified as one species with *B. mycoides* (Liu et al., 2018) – and the thermotolerant and cytotoxic *B. cytotoxicus* (Guinebretière et al., 2013). Diverse classification methods have been proposed over the years (for a recent review, refer to Carroll et al., 2021) in an effort to resolve the ongoing taxonomic conflicts surrounding *B. cereus s.l.* due to (i) an insufficient differentiation at the genomic level and (ii) whether or not plasmids and MGEs should be included in the phylogenetic analyses. Members of *B. cereus s.l.* carry a wide range of plasmids, some of which are susceptible candidates for horizontal dissemination (Kolstø et al., 2009; Yuan et al., 2010). Although many studies presented evidence that MGEs and HGT can confound the phylogenetic process (Bazinet, 2017), there is no denying the importance of extra-chromosomal entities in defining a species’ lifestyle, hence their importance in the phylogenetic process (Liu et al., 2015; Patiño-Navarrete and Sanchis, 2017).

In fact, the three main members of the *B. cereus* group are discriminated based on specific toxins they secrete, whose genetic determinants are often encoded on large transmissible plasmids, or on specific plasmids they carry. For instance, *B. anthracis* is characterized by two large plasmids, pXO1 and pXO2, responsible for the production of the bipartite anthrax toxins and the anthrax capsule, respectively (Baillie and Read, 2001). Both pXO1 and pXO2 encode genes reminiscent of conjugation systems, but they show no ability for self-transfer, although they were proven as mobilizable (Grynberg et al., 2007). Other plasmids share the backbone of pXO1 and pXO2 and are therefore grouped into distinct plasmid families. pXO2-like plasmids are likely to depict self-transmissible capacities as some of their representatives (i.e., pAW63 from *B. thuringiensis* sv. *kurstaki*, pBT9727 from *B. thuringiensis* sv. *konkukian* and pXO2 from *B. anthracis*) harbor a *tra* (*tra*nsfer) region with genes homologous to the main components of functional conjugative systems and T4SS. While both pAW63 and pBT9727 are effective conjugative plasmids, pXO2 has an interrupted *virD4*-like gene likely responsible for its inability to conjugate autonomously (Van der Auwera et al., 2005; Hu et al., 2009b; see Section “pAW63 and other pXO2-like plasmids”).

The pXO1-like plasmid family includes pCER270, a plasmid bearing the genetic determinants of the cereulide, a dodecadepsipeptide responsible for the emetic syndrome in *B. cereus*-mediated food infections (Hoton et al., 2005; Rasko et al., 2007). Other pXO1-like plasmids are pBMB67 from *B. thuringiensis* (Chao et al., 2007), pER272 and pBC10987 from *B. cereus* strains (Rasko et al., 2007; Mei et al., 2014) and pBCXO1 from *B. cereus* G9241 and *B. cereus* biovar *anthracis* (Hoffmaster et al., 2004; Brézillon et al., 2015). These plasmids share a high degree of synteny with pXO1 but lack or diverge in their pathogenicity genetic determinants. Contrary to the pXO2-like plasmids, none of the pXO1-like plasmids harbor self-transfer features, although pBMB67 displays CDS (Coding DNA Sequence) homologous to the VirB/D4 model for T4SS and was therefore described as a putative conjugative plasmid (Chao et al., 2007).

The main feature associated with *B. thuringiensis* is the production of entomopathogen delta-endotoxins (Cry toxins) in parasporal crystals, used worldwide in biopesticide preparations (Bravo et al., 2007; Palma et al., 2014). The genetic determinants of Cry toxins are plasmid-borne, encoded on *cry* plasmids, which are either transmissible upon mobilization, like pBtoxis from *B. thuringiensis* sv. *israelensis* (Berry et al., 2002; Hu et al., 2005), or conjugative themselves, like pHT73 from *B. thuringiensis* sv. *kurstaki* (Wilcks et al., 1998). Establishing a link between the presence of plasmids and the production of Cry toxins was at the base of the discovery of conjugation-like events between strains of *B. thuringiensis* (see Section “First hints of conjugation and premises of a new transfer tool”).

Besides toxin plasmids, *B. cereus s.l.* harbors a large pool of plasmids and other mobile elements. While plasmids typically promote intercellular genetic moves, other MGEs, like insertion sequences (IS) and transposable elements (Tn), are usually responsible for intracellular mobility, i.e., moving from one location to another within the same genome. Transposable elements are for instance more prevalent in toxin plasmids of *B. thuringiensis*, namely about 30% of the total “*thuringiensis*” plasmid pool, as they often flank *cry* genes (Fiedoruk et al., 2017; Fayad et al., 2019, 2021a). In natural environments, *B. cereus s.l.* isolates also present a flexible pool of extrachromosomal molecules, with HGT evidenced between closely related isolates (Hu et al., 2009a). HGT within the *B. cereus* group is not restricted to plasmids. Some chromosomally-encoded enterotoxin operons (*hbl* and *cytK*) of *B. cereus* were for example shown to have spread by HGT amongst different taxonomic clusters (Böhm et al., 2015). Although the cereulide genetic determinants (*ces*) are located on non-self-transmissible plasmids or in the chromosome, they are also prone to horizontal dissemination because they are typically flanked by MGEs, like Tn*ces* in emetic strains of *B. weihenstephanensis* (Mei et al., 2014).

With their multiple combinations of plasmids and other MGEs, strains of *B. cereus s.l.* are provided with a strong potential for adaptability. Indeed, HGT is recognized as a major route for acquiring new potentially beneficial genetic material, enabling bacteria to adapt and migrate to new environmental niches (McInerney et al., 2017), and the *B. cereus* group makes no exception. However, acquiring foreign DNA can sometimes be harmful to bacteria thriving in some habitats. Therefore, bacteria developed protection systems preventing from such invasion, such as the antiviral CRISPR-Cas system (Barrangou and Marraffini, 2014). In staphylococci, the immune and protective function of the CRISPR-Cas system acts as barriers limiting the acquisition of MGEs by HGT (Marraffini and Sontheimer, 2008). Interestingly, CRISPR-Cas systems are less prevalent and mostly inactivated in *B. cereus s.l.* (Zheng et al., 2020). In addition, strains of *B. cereus* with functional CRISPR-Cas systems showed a restricted ecological niche, a poorer pool of MGEs and an impaired adaptation to harsh environments. On an evolutionary point of view, acquisition of MGEs and ecological adaptability were apparently favored by *B. cereus s.l.*, rather than protective CRISPR-Cas systems (Zheng et al., 2020), further illustrating the importance of HGT within this specific bacterial group.

## Historical accounts

**Figure 1:**
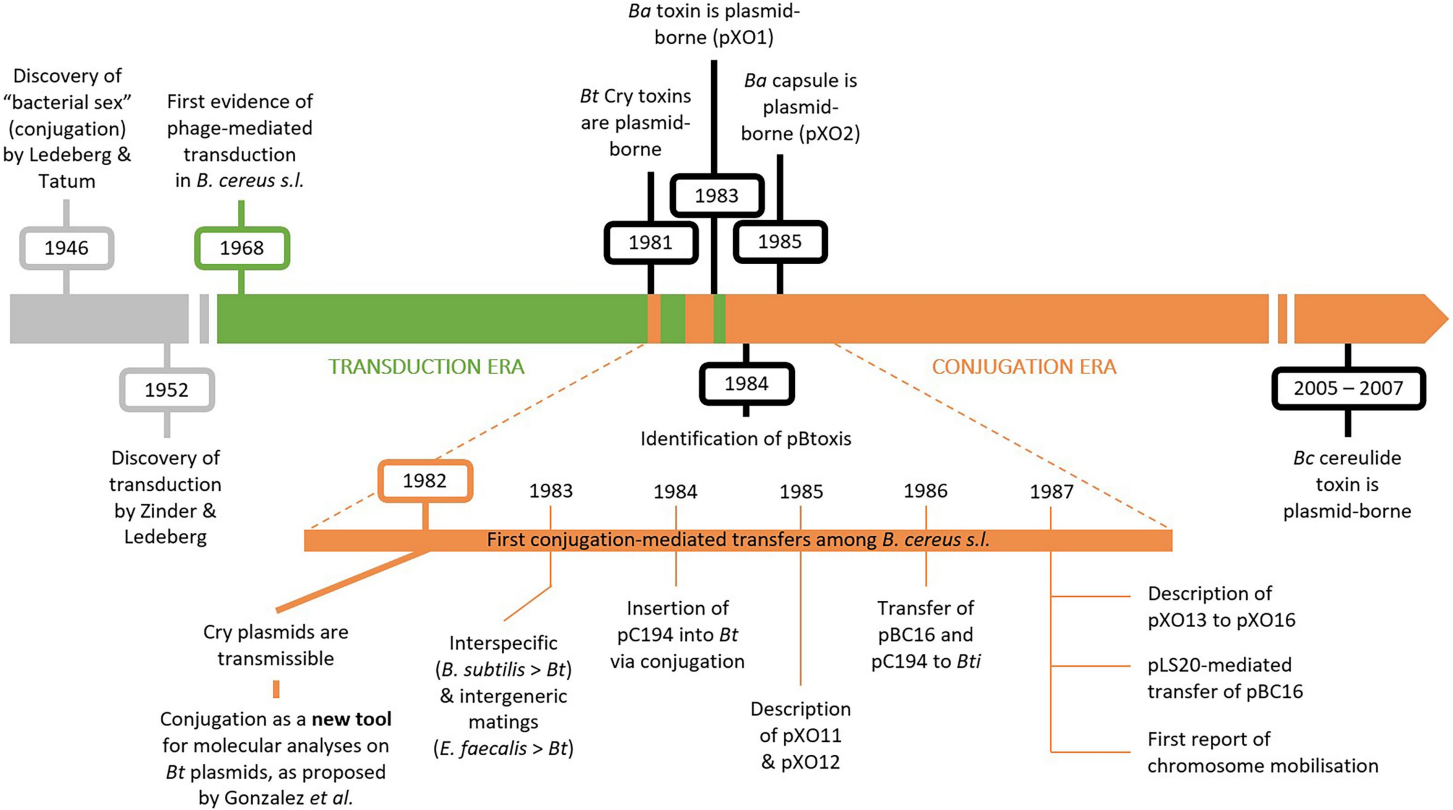
Timeline displaying milestones in *Bacillus cereus s.l*. molecular research. In the 1960’s and 1970’s, molecular research in *B. cereus s.l.* was mostly conducted using phage-mediated transduction (“transduction era” in green). Since the discovery of conjugative transfer in *B. cereus s.l.*, plasmid-mediated conjugation became the prevalent technique (“conjugation era” in orange). Plasmid-borne toxins play a key role in *B. cereus s.l.* taxonomy and were mostly identified alongside conjugation (in black). The historical discovery of conjugation and transduction are depicted in grey. *Ba*, *B. anthracis*; *Bt*, *B. thuringiensis*; *Bc*, *B. cereus*; *Bti*, *B. thuringiensis* sv. *israelensis.*

Up until the discovery of conjugative transfer among the *B. cereus* group in the early 1980s, phage-mediated transduction was the main technique available for studying *B. cereus s.l.* at a molecular level (Thorne, 1978). Most early molecular studies focused on *B. thuringiensis* due to its entomocidal Cry toxins and their common application as biopesticide (for a review, see Palma et al., 2014). The historical milestones described in this section are illustrated on Figure 1.

### *Bacillus cereus s.l.* in the pre-conjugation era

Transducing phages were mostly used for genetic experiments in *B. cereus s.l.* during the 60’s and 70’s, before the discovery of conjugative plasmids and their application as molecular tools (Figure. 1). The first evidence of phage-mediated transduction in *B. cereus s.l.* dates back to 1968, when a transducing phage, designated CP51, was isolated from a soil sample along with its host bacterium *B. cereus* strain 569 (Thorne, 1968). Phage CP51 was later shown to successfully mediate the transfer of the plasmidial vectors pBC16, conferring resistance to tetracycline (Tet^R^), and pC194, conferring resistance to chloramphenicol (Cm^R^), to specific strains of *B. cereus*, *B. anthracis* and *B. thuringiensis* (Ruhfel et al., 1984) and therefore became a tool for genetic manipulations in *B. cereus s.l.* Other CP-phages mediating generalized transduction in *B. cereus s.l.* were also described, such as CP53, stable at lower temperatures (Yelton and Thorne, 1970), CP54, displaying a broader host spectrum (Thorne, 1978) and CP54-Ber, isolated from CP-54 lysates and infecting *B. thuringiensis* sv. *berliner* strain 1715 (Lecadet et al., 1980). Many non CP-phages were also described for their potential in generalized transduction in the *B. cereus* group (for extensive reviews, refer to Gillis and Mahillon, 2014 or Sorokin, 2012).

Transducing phages of *B. cereus s.l.* are grouped into three categories, based on their size and host range (Sorokin, 2012). A restricted host spectrum characterizes smaller phages. Medium-size phages show rather poor transduction efficiency, although they include CP51, the most-predominantly used phage for transduction experiments in *B. cereus s.l.* (Thorne, 1978). Large phages exhibit broader host spectra and were good candidates for early genome mapping experiments. For instance, the genome of *B. thuringiensis* strain 4042B was mapped using the phages TP13 and TP18 (Barsomian et al., 1984). Given its insecticidal properties, *B. thuringiensis* was the main focus of most transduction-based studies in the *B. cereus* group (Sorokin, 2012).

### First hints of conjugation and premises of a new transfer tool

Forty years ago, González and co-workers reported, for the first time, a conjugation-like process between strains of *B. thuringiensis* during growth in mixed cultures, while studying the genetic relationship between the presence of plasmids and the production of delta-endotoxin crystals. Notably, the plasmid-borne origin of *B. thuringiensis* delta-endotoxins was shown, along with the transmissible nature of the “*cry* plasmids” (González et al., 1981, 1982). The transfer of a *cry* plasmid was also observed between strains of *B. thuringiensis* and *B. cereus s.s.* This conjugation-like event consisted in the transfer of a *cry* plasmid accompanied by the transfer of a cryptic conjugative plasmid, namely a mobilization event (González et al., 1981, 1982). It is important to note that Cry toxins, and therefore *cry* plasmids, were, and still often are, considered a major feature distinguishing *B. thuringiensis* from other *B. cereus s.l.* strains. Thus, the transmissibility of *cry* plasmids reinforced the running hypothesis that both species were closely related (González et al., 1982) and further blurred the lines of species distinction among members of the *B. cereus* group.

One of the obstacles in the study of *B. thuringiensis* genetics has for long been the lack of effective transformation tools. The newly described conjugation-based system provided researchers with a potential new “transformation” system for studying *B. thuringiensis* plasmids (Figure 1; González and Carlton, 1982). The examples mentioned hereinafter rely on mobilization events, rather than on conjugative transfers *per se*. Conjugative plasmids are usually naturally cryptic and transfers were followed by assessing the transmissibility of detectable mobilizable plasmids, like *cry* plasmids or small plasmids encoding an antibiotic resistance marker.

The newly proposed transfer tool was used to transfer the *cry* genes of *B. thuringiensis* sv. *berliner* 1715, cloned firstly into *B. subtilis*, to an acrystalliferous strain of *B. thuringiensis* sv. *kurstaki* and an “*israelensis*” strain. The acrystalliferous strain could then produce toxin crystals and the “*israelensis*” strain could therefore produce two types of delta-endotoxins, broadening its range of susceptible target insects (Klier et al., 1983). Besides interspecies matings between *B. subtilis* and *B. thuringiensis*, pAM*β*1 was successfully exchanged in intergeneric matings between *Streptococcus* (*Enterococcus*) *faecalis* and *B. thuringiensis* (Lereclus et al., 1983, 1985). The conjugation-mediated transfer tool also served to identify a large 75-MDa transmissible plasmid – later named pBtoxis – responsible for producing Cry toxins in the mosquitocidal *B. thuringiensis* sv. *israelensis* (González and Carlton, 1984). The Cm^R^ plasmid pC194 and the Tet^R^ plasmid pBC16 were also successfully introduced into strains of *B. thuringiensis* by mobilization (Fischer et al., 1984; Loprasert et al., 1986). Other interspecies matings were later performed between *B. subtilis* and *B. cereus s.l.*, where the transfer of pBC16 was mediated by the *B. subtilis* conjugative plasmid pLS20 (Koehler and Thorne, 1987).

In parallel, the transfer features of the large plasmids pXO11, pXO12, pXO13, pXO14, pXO15 and pXO16 originating from different serovars of *B. thuringiensis* were investigated by following the mobilization of pBC16 (Battisti et al., 1985; Reddy et al., 1987). All six plasmids could successfully be transferred to *B. cereus s.s.* and *B. anthracis*, except for pXO15, which was only transferred to *B. cereus s.s.* Because pXO12 carries Cry toxin determinants, its conjugation-mediated transfer resulted in transcipients with “hybrid” phenotypes, as *B. cereus s.s.* and *B. anthracis* transcipients could produce Cry toxins besides their characteristic “*cereus*” and “*anthracis*” toxins (Battisti et al., 1985). On the contrary, no function other than conjugation could characterize the five other plasmids (Reddy et al., 1987). With their ability to transfer a large range of plasmids from their respective donor strains, plasmids pXO11 to pXO16 were useful exchange tools for analyzing genetic determinants on plasmids of *B. anthracis*, *B. cereus* and *B. thuringiensis* (Battisti et al., 1985). For instance, both pXO12 and pXO14 successfully triggered the transfer of the “*anthracis*” plasmids pXO1 and pXO2 to plasmid-cured *B. anthracis* and *B. cereus*, while pXO16 was only capable of transferring pXO2, and pXO13 could mobilize none (Battisti et al., 1985; Reddy et al., 1987). In the case of pXO12-mediated mobilization of pXO1 and pXO2, the specific mobilization process is called conduction. Conduction usually calls for recombination events either between homologous or site-specific sequences, or mediated by transposable elements on one of the plasmids involved (Andrup, 1998). Here, cointegrates of pXO12 and pXO1 (or pXO2) were observed, and the recombination events were most likely mediated by the pXO12-encoded transposon Tn*4430* (Green et al., 1989). In opposition to conduction, pXO12 mediated the transfer of the small plasmid pBC16 by donation, a process whereby both plasmids remain physically distinct and do not associate (Green et al., 1989; Andrup, 1998).

Chromosomal fragments were also discovered to transfer laterally at low frequencies alongside the transfer of a conjugative plasmid, by a mobilization-like mechanism (Aronson and Beckman, 1987). Chromosome mobilization was first noticed between strains of *B. thuringiensis* and *B. cereus s.s.*, invariably accompanied by the transfer of at least one plasmid from the donor strain. The transfer efficiency of chromosomal fragments was reduced compared to the transfer efficiency of plasmids. The efficiency also varied for different chromosomal markers, suggesting that the localization of the marker could impact the transfer efficacy (Aronson and Beckman, 1987). Then, a study focusing on plasmid and chromosome transfers between *B. thuringiensis* sv. *israelensis* and various *B. thuringiensis* serovars showed that the transfer frequencies varied depending on the recipient strain and on the transferred plasmid (Wiwat et al., 1990). While transfer frequencies of chromosomal fragments using broth mating technique were very low, it was suggested that filter mating might improve such frequencies (Wiwat et al., 1990). With current knowledge, the multiple mobilization events witnessed in *B. thuringiensis* sv. *israelensis* can most likely be attributable to pXO16. With that, researchers’ observations of chromosome transfer and suggestion about improving transfer frequencies by filter mating were later confirmed (Makart et al., 2017, 2018; Hinnekens et al., 2019; see Section “pXO16, the unique conjugative master”).

Circling back to toxin-carrying plasmids, establishing the connection between plasmids and *cry* genes in *B. thuringiensis* opened the possibility about the anthrax toxin genes being plasmid-borne (Mikesell et al., 1983). The genetic determinants of both the protective antigen, i.e., one of the three proteins forming the anthrax bipartite toxins, and the anthrax capsule were indeed demonstrated to be encoded on two distinct large plasmids, pXO1 (Vodkin and Leppla, 1983) and pXO2 (Green et al., 1985), respectively (Figure 1). Isolating and characterizing strains of *B. anthracis* carrying both plasmids led to the conclusion that both pXO1 and pXO2 are involved in the pathogenicity and the virulence of *B. anthracis* (Uchida et al., 1985, 1986).

## Conjugative plasmids of *Bacillus cereus s.l.*

Over the years, several plasmids from strains of *B. cereus s.l.* were characterized as conjugative, based either on experimental transfer evidence or on strong bioinformatics predictions (Table 1). Although recent works keep focusing on the importance of plasmids in the *B. cereus* group, especially for *B. thuringiensis* and its Cry toxins (Bolotin et al., 2017; Gillis et al., 2018; Fayad et al., 2019, 2021a; Zheng et al., 2020), few studies have investigated conjugative transfer *per se*. Furthermore, reported transfer events often consist in mobilization events without incriminating the implicated conjugative plasmid (Fguira et al., 2014). Therefore, only 20 conjugative plasmids of *B. cereus s.l.* have been reported to date, but the conjugative capacities of eight of them lack experimental proofs (Table 1).

**Table 1:** Conjugative plasmids from *B. cereus s.l.* List of *B. cereus s.l.* plasmids reported as conjugative – or potentially conjugative – and their main characteristics.

Plasmid	Original host	Size (kb)	GenBank accession no.	Features	Type replicon	Experimental proof of conjugation	Source
pXO11, pXO13-pXO15	*B. thuringiensis*	–	–	First reported conjugative plasmids from *B. thuringiensis*	–	Yes	Battisti et al. (1985), Reddy et al. (1987)
pXO12	*B. thuringiensis* sv. *thuringiensis* 4042A	112.5	–	Cry plasmid; one of the first reported conjugative plasmids from *B. thuringiensis*	–	Yes	Battisti et al. (1985)
pGB130	*B. cereus* 5	–	–	Mercury resistance	–	Yes	Belliveau and Trevors (1990)
pXO16	*B. thuringiensis* sv. *israelensis*	349.6	CP003764.1	Aggregation phenotype; novel T4SS	–	Yes	Reddy et al. (1987)
pAW63	*B. thuringiensis* sv. *kurstaki*	71.8	DQ025752.1	*tra* region with homologs to the VirB/D4 model, pXO2-like family	pAM*β*1 family	Yes	Wilcks et al. (1998)
pBT9727	*B. thuringiensis* sv. *konkukian*	77.1	NC_006578.1	Instability in heterologous genomic background, pXO2-like family	pAM*β*1 family	Yes	Van der Auwera et al. (2008)
pHT73	*B. thuringiensis* sv. *kurstaki* HD73	77.3	NC_020249.1	Cry plasmid (*cry1Ac* gene), *tra* region	*ori44*	Yes	Wilcks et al. (1998)
pFR55	*B. thuringiensis* INTA-FR7-4	55.7	EU362919.1	*tra* region with homologs to the VirB/D4 model & similar to pAW63 *tra* region	*ori44*	No	Amadio et al. (2009)
pBMB67	*B. thuringiensis* YBT-1520	67.1	NC_009841.1	Modular organization, including one module with homology to VirB/D4 model	-	No	Chao et al. (2007)
pIS56-63	*B. thuringiensis* sv. *thuringiensis* IS5056	63.8	NC_020392.1	Cry plasmid (*cry1Ab21* gene), *tra* region almost identical to pHT73 *tra* region	*ori44*	No	Murawska et al. (2014)
pBMB76	*B. thuringiensis* sv. *tenebrionis* 4AA1	76.9	NZ_CP010580.1	–	*ori60*	Yes	Hu et al. (2020)
pBMB68	*B. thuringiensis* sv. *tenebrionis* 4AA1	68.4	NZ_CP010581.1	Putative conjugative region	*ori43*	No	Hu et al. (2020)
pBMB232	*B. thuringiensis* sv. *tenebrionis* 4AA1	233	NZ_CP010578.1	Putative conjugative region	*orf 156/157*	No	Hu et al. (2020)
pLUSID1	*B. thuringiensis* HER1410	368.2	NZ_CP050184.1	Putative conjugative region around *ftsZ*-like gene	–	No	Lechuga et al. (2020)
pE81-53	*B. cytotoxicus* E8.1	53.1	NZ_CP066195.1	Putative conjugative region	–	Yes	Fayad et al. (2021b)
pE81-84	*B. cytotoxicus* E8.1	83.6	NZ_CP066196.1	Putative conjugative region, distantly related to pXO2-like plasmids	–	No	Fayad et al. (2021b)
pE283-80	*B. cytotoxicus* E28-3	79.7	NZ_CP066190.1	Putative conjugative region, distantly related to pXO2-like plasmids	–	No	Fayad et al. (2021b)

The first plasmids described as conjugative, i.e., pXO11 to pXO15, were presented in Section “First hints of conjugation and premises of a new transfer tool.” In this section, the more recently reported *B. cereus s.l.* conjugative elements have been split into three groups for readily description: (i) the conjugative plasmids sharing the *tra* region of pAW63 and the pXO2-like plasmids, (ii) the unique conjugative system of pXO16, and (iii) other major conjugative plasmids, including pHT73 and plasmids sharing an *ori44*-like replicon.

### pAW63 and other pXO2-like plasmids

#### The broad-host range plasmid pAW63

*B. thuringiensis* sv. *kurstaki* HD73, a common commercial biopesticide strain, was part of González and colleagues’ original study in early 1980s that discovered the transmissible nature of *cry* plasmids (González et al., 1981, 1982). This “*kurstaki*” strain was later shown to contain a second large plasmid, dubbed pAW63, aside from the *cry* plasmid pHT73 (Wilcks et al., 1998; see Section “Ori44-type replicon related plasmids”). This plasmid exhibited high transfer frequencies, reaching up to 100% efficiency between “*kurstaki*” strains in liquid mating, reminiscent of pXO16 transfer efficiency between strains of *B. thuringiensis* sv. *israelensis* (Wilcks et al., 1998; Van der Auwera et al., 2005; see next Section). pAW63 was classified as a broad host range plasmid, like the plasmid pIP501 initially isolated from *Streptococcus* (Evans and Macrina, 1983). It could indeed transfer to *B. thuringiensis* sv. *israelensis*, *B. cereus*, *Bacillus licheniformis*, *B. subtilis* and *Bacillus* (*Lysinibacillus*) *sphaericus* (Wilcks et al., 1998), as well as to more distantly-related species, such as *Listeria innocua* and *E. faecalis* (Van der Auwera et al., 2005). Studies also assessed pAW63 transfer efficiency in food matrices, especially dairy products and plant-based drinks (Van der Auwera and Mahillon, 2008; Modrie et al., 2010; see Section “Conjugation in foodstuffs”).

**Figure 2:**
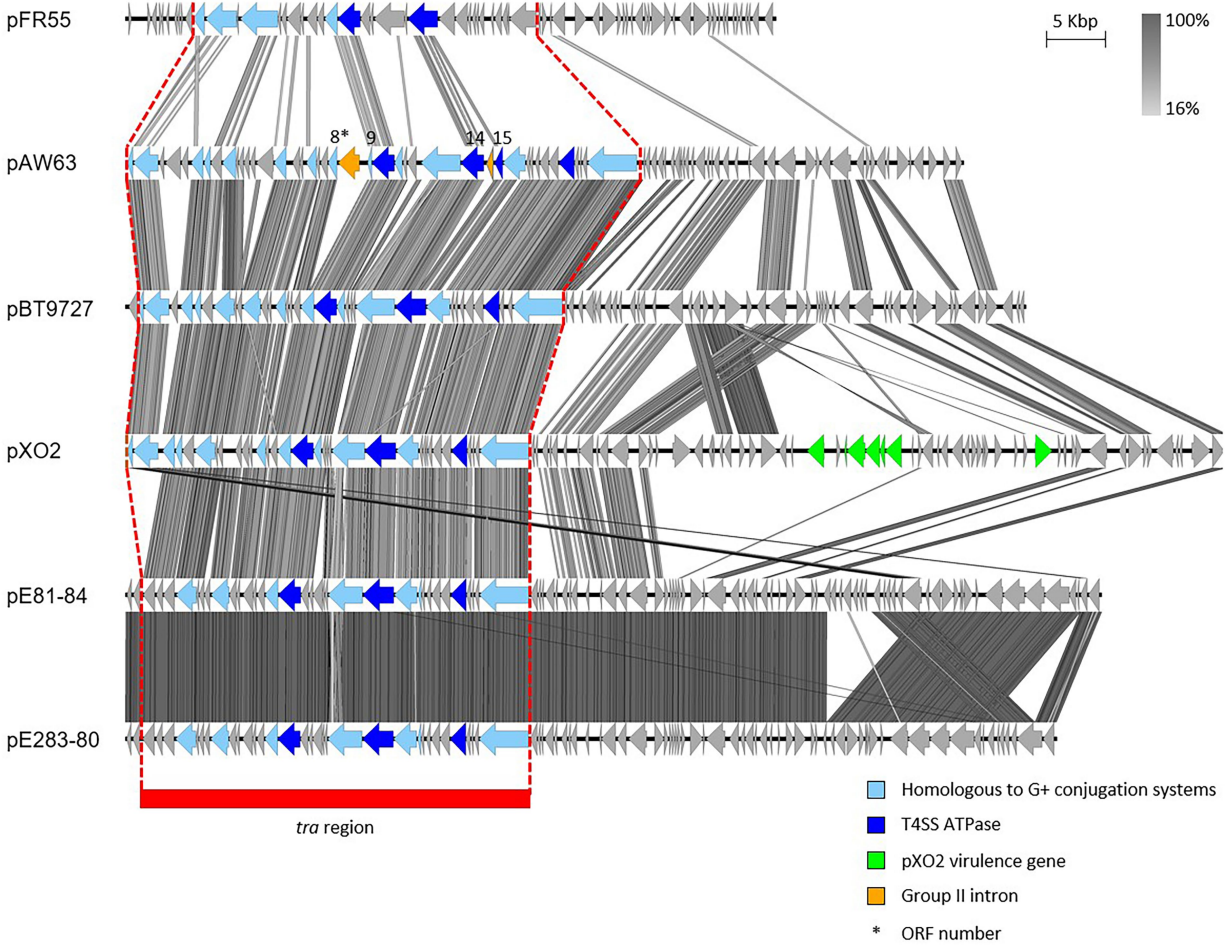
Sequence comparison of the (putative) conjugative plasmids of the pXO2-like family. The *tra* region of the plasmids pFR55, pAW63, pBT9727, pXO2, pE81-84 and pE283-80 share evident similarities. CDS depicted in light blue share homologies with other known conjugative systems; CDS depicted in dark blue are the putative ATPases; both CDS in orange in pAW63 sequence correspond to group II introns interrupting the genes encoded by the CDS 8–9 and 14–15; CDS colored in green encode Cap and Acp virulence factors of pXO2. The region highlighted in red corresponds to the *tra* region. The image was generated by Easyfig 2.0 software. Genbank accession numbers: pXO2: NC_007323.3; others: Table 1.

Studying pAW63 4.1-kb replicon highlighted a strong similarity between pAW63 largest replication protein, Rep63A, and the replication proteins RepR and RepE from pIP501 and pAM*β*1, respectively (Wilcks et al., 1999). Both pIP501 and pAM*β*1 belong to the pAM*β*1-family of theta replicating plasmids, whose replication relies on the host DNA polymerase 1 and is single-stranded DNA-independent. As it shares these features, pAW63 was associated within the same plasmid family (Wilcks et al., 1999). The full sequence of pAW63 was published in 2005 and revealed a modular organization. One 42-kb module forms the *tra* region and encodes 15 CDSs putatively assigned to conjugative functions. Among these, three CDSs share homologies with the ATPases from the Vir secretion system, the CP VirD4, the motor ATPase VirB4 and the ATPase VirB11. Other CDSs share homologies with conjugation proteins from various Gram-positive T4SS (Van der Auwera et al., 2005). Interestingly, two group II-related introns interrupt two genes within the conjugative locus (Figure 2). On the one hand, ORFs 8 and 9 are split by the B.th.I1 intron and are predicted to encode the conjugative cell wall hydrolase. On the other hand, ORFs 14 and 15 are separated by the B.th.I2 intron but form one functional gene encoding the putative VirD4-like CP (Van der Auwera and Mahillon, 2008). Group II introns have also been found on the conjugative plasmid pRS01 from *Lactococcus lactis* (Belhocine et al., 2005) and on other plasmids from *B. cereus s.l.* (Tourasse et al., 2005). It is worth mentioning that most of the current knowledge on pAW63 *tra* region and associated conjugation mechanism was inferred from homologies with other T4SS and does not rely on experimental data.

#### The family of pXO2-like plasmids

Analyzing pAW63 sequence indicated strong similarities with the “*anthracis*” plasmid pXO2 from *B. anthracis* and the pBT9727 plasmid from strain 97–27 of *B. thuringiensis* sv. *konkukian* (Van der Auwera et al., 2005). Indeed, among the 76 CDSs predicted on pAW63, 42 are common between the three plasmids, with 50 CDSs shared by pAW63 and pXO2 and 49 by pAW63 and pBT9727. Of specific interest is the high conservation of the region corresponding to the pAW63 transfer module in pXO2 and pBT9727 (Figure 2; Van der Auwera et al., 2005). Although pXO2 is mobilizable but non conjugative, pBT9727 was shown to promote its own transfer and to trigger the mobilization of small co-resident mobilizable plasmids (Van der Auwera et al., 2008). Based on comparative sequence analyses, all three plasmids were proposed to derive from a same ancestral conjugative plasmid, from which pAW63 and pBT9727 retained conjugation capacities, while pXO2 genetically drifted and lost the potential to conjugate (Van der Auwera et al., 2008). Most constituents of the *tra* region are intact in pXO2 compared to pAW63 and pBT9727. However, at least two putatively major elements for conjugation display punctual mutations in pXO2, including the *virD4*-like gene, presumably leading to frameshifts and disruption of the conjugative functions (Van der Auwera et al., 2005).

pXO2, pAW63 and pBT9727 compose the core of the pXO2-like plasmid family, but other conjugative – or putatively conjugative – plasmids also share some similarities. Three closely related plasmids from *B. cytotoxicus* were recently described as distantly related to the trio of pXO2-like plasmids (Fayad et al., 2021b). Two of them, pE81-84 and pE283-80, also harbor a region reminiscent of the *tra* region of pAW63 and kin, suggesting a conjugative potential for both plasmids. As illustrated on Figure 2, these two plasmids are highly similar, and a large portion of their sequence is very similar to that of pXO2. The putative conjugative region encodes three candidates for ATPase functions, including one putative CP, as well as candidates for transglycosylase and relaxase functions. Although they harbor all signature elements of a functional T4SS, experimental data are lacking to confirm the conjugative nature of pE81-84 and pE283-80.

pFR55, a plasmid isolated from *B. thuringiensis* INTA-FR7-4, presents a *tra* region with multiple CDSs sharing similarities to CDSs involved in pXO2-like plasmid conjugation (Figure 2; Amadio et al., 2009). pFR55 putative conjugative locus encodes a relaxase, a transglycosylase, a putative CP and an ATPase, all key elements of functional conjugative systems (Goessweiner-Mohr et al., 2013). Given these characteristics, pFR55 has a strong potential for conjugation, but experimental evidence of its self-transferability is lacking. Apart from its *tra* region, pFR55 does not share the common backbone of pXO2-like plasmids (Figure 2). For instance, its replication region belongs to the family of *ori44-*type replicon, like the well-studied *cry* plasmid pHT73 from *B. thuringiensis* sv. *kurstaki* HD73 (Amadio et al., 2009), and pFR55 forms therefore a separate group from pXO2-like plasmids.

#### Prevalence of pXO2-like plasmids

As pXO2-like transfer modules exhibit a significant degree of conservation amongst several described conjugative plasmids, the prevalence of such module was evaluated at a larger scale (Hu et al., 2009a, 2009b). pXO2-like plasmids/replicons were widespread in environmental isolates and had therefore a significant potential as conjugative plasmids. However, the proportion of strains demonstrating conjugative elements remained minor compared to the initial population of interest, especially from environmental samples. Indeed, in two independent studies, pXO2-like transfer modules were detected in only 5% of the *B. cereus s.l.* strains selected from both environmental samples and laboratory collections (Hu et al., 2009a, 2009b).

### pXO16, the unique conjugative master

The conjugative plasmid pXO16, first identified by Reddy et al. in 1987, was thoroughly investigated by different research teams. Much attention was given to its phenotypic description, mainly for the aggregation phenotype induced by pXO16, as well as to the genetic components underlying its conjugation mechanism.

#### Aggregation-mediated conjugation

Aggregation-mediated conjugation mechanisms have been reported for various conjugative systems. The most studied example is the conjugative system of *E. faecalis*, which involves clumping of donor cells under the influence of a recipient-borne sex pheromone (Dunny et al., 1978). Cell aggregates also mediate plasmid transfer in conjugative systems of some lactic bacteria, such as *L. lactis* and *Lactobacillus plantarum*. In *L. lactis*, the aggregation-inducing factor is actually chromosomally encoded (Walsh and McKay, 1981; Van Der Lelie et al., 1991), while the aggregation-mediated conjugation system of *L. plantarum* is neither plasmid-encoded nor specific and facilitates the formation of mating pairs for various conjugative plasmids (Reniero et al., 1992). Macroscopic cell aggregates were also witnessed in broth matings of *B. thuringiensis* sv. *israelensis* and were attributed to pXO16 conjugation (Andrup et al., 1993; Jensen et al., 1995). pXO16-mediated aggregation was thoroughly studied and brought to light some key features of pXO16 conjugative system, e.g., the transmissibility of the aggregation phenotype alongside pXO16 transfer (Jensen et al., 1996), the mobilization of small co-resident plasmids (Andrup et al., 1996), and the importance of S-layer proteins in transfer efficiency (Wiwat et al., 1995).

The interest for pXO16-mediated aggregation got renewed when the aggregation-encoding genes were localized within a 25-kb *agr* region of pXO16 sequence (Makart et al., 2015). After over two decades of hypothesizing that aggregation was a prerequisite for pXO16 transfer, an aggregation-deficient mutant was constructed and exhibited reduced, yet effective, transfer in absence of the aggregation proteins (Makart et al., 2018). Thus, pXO16 aggregation facilitates its transfer but is not required. By studying *B. thuringiensis* sv. *israelensis* aggregation under atomic force microscopy (AFM), surface proteins encoded in the *agr* region were shown to mediate specific interactions between mating partners, resulting in strong adhesion forces (~2nN; Feuillie et al., 2018).

#### Kinetics of transfer and DNA capture properties

pXO16 transfer is characterized by high-speed, as transfer is completed in *ca.* 3.5–4 min, by the formation of mating pairs involving one donor and one recipient within large mating aggregates, and by the hypothetical low copy number of the plasmid (Andrup et al., 1998). Primary transfer of pXO16 can be detected within 10 min and peaks at 40–50 min after mating onset (Timmery et al., 2009). A 10-min recovery period is required in between two transfer events from a same donor, and a 40 min maturation time is necessary for newly formed transconjugants to turn into donors themselves (Andrup et al., 1998).

Back in 1987, pXO16 was first reported for triggering the transfer of the mobilizable plasmid pBC16 by a conjugation-like event between *B. thuringiensis* sv. *israelensis* and strains of *B. cereus s.s.* or *B. anthracis* (Reddy et al., 1987). Further work dived into pXO16 mobilization capacities and revealed that pXO16 can mobilize mobilizable – i.e. usually encoding an *oriT* and a relaxase, but no T4SS – as well as “non-mobilizable” – i.e. lacking all elements necessary for conjugation (*oriT*, relaxase and T4SS) – co-resident plasmids (Timmery et al., 2009). It can also trigger the capture of a plasmid from a recipient cell to the pXO16-harboring donor cell *via* a mechanism known as retro-mobilization (Timmery et al., 2009). More recently, studies reported that pXO16 could trigger the transfer of chromosomal loci between mating partners (Makart et al., 2017). Furthermore, chromosomal fragments of at least 791 kb could be transferred between strains of *B. thuringiensis* sv. *israelensis* and chromosomal markers were successfully transferred by pXO16 between strains of *B. cytotoxicus* (Makart et al., 2018; Koné et al., 2021). pXO16 transfer and capture capacities were also evaluated under different ecological and food-related conditions, such as the midgut of dipteran larvae or milk and rice pudding (Thomas et al., 2001; Van der Auwera et al., 2007; see Sections “Conjugation under ‘natural’ environmental conditions” and “Conjugation in foodstuffs”).

#### Conjugation apparatus, a novel type of T4SS

Despite detailed work on phenotypical features of pXO16 transfer, little was known about the genetics underlying its conjugation mechanism until pXO16 sequence was analyzed in 2015 (Makart et al., 2015). Analysis of the 434 CDSs, covering more than 85% of the 350-kb sequence, revealed very few relevant homologies with public databases. Among the few CDSs with a predicted functional annotation, none were related to any T4SS components identified so far, suggesting that pXO16 conjugative system is rather unique (Makart et al., 2015). Based on detailed bioinformatics studies and on experimental data, a 25-kb locus involved in conjugation was delimitated and referred to as the *transfer of israelensis plasmid* (*tip*) locus (Makart et al., 2018). Within the 16 CDSs forming the *tip* locus, genes encoding essential conjugative functions were identified, such as two ATPases and one cell wall hydrolase (Makart et al., 2018; Hinnekens et al., 2021). While these functions are widely recognized as essential in canonical T4SS, key elements, particularly the relaxase and the *oriT*, remain unidentified in pXO16 conjugative apparatus.

#### Transfer of pXO16 across the *Bacillus cereus* group

From first investigations, pXO16 host spectrum was thought to be rather restricted, with only few serovars of *B. thuringiensis* and few strains of *B. cereus s.s.* capable of receiving pXO16 (Jensen et al., 1996). These conclusions were based on the hypothesis that aggregation (Agr) was mandatory for the conjugation-mediated transfer of pXO16. Strains carrying pXO16, designated Agr^+^, could form macroscopic aggregates with some other strains (Agr^−^ strains). Only those Agr^−^ strains were considered as potential recipients for pXO16 (Table 2), while strains which could not form macroscopic aggregates with an Agr^+^ strain were ignored (Jensen et al., 1996). However, as indicated above, an aggregation-deficient variant of pXO16 was capable of conjugative transfer, albeit at dramatically reduced frequencies, indicating that the Agr phenotype is important but not mandatory for pXO16 transfer, as previously stated (Makart et al., 2018). Based on this new finding, previously overlooked non-aggregating strains of *B. cereus s.l.* were reported to successfully act as recipients for pXO16 transfer in liquid matings (Makart et al., 2018). Further work demonstrated that mimicking Agr by using solid mating conditions could compensate Agr deficiency and allow transfer to several *B. cereus s.l.* strains, including *B. cytotoxicus*, *B. mycoides* and strains appertaining to the same clade of *B. anthracis* but lacking the “*anthracis*” virulence genes (Hinnekens et al., 2019; Hinnekens and Mahillon, 2022; Table 2). Based on recent host spectrum data, assuming pXO16 transfer based on the Agr phenotype of the recipient strain is no longer relevant. Similarly, the Agr phenotype should not be confused with the wild-type or aggregation-deficient version of pXO16, as they, respectively, express strain-specific and plasmid-specific features.

**Table 2:** pXO16 host spectrum. List of all the strains to which pXO16 transfer was successfully demonstrated under liquid or solid mating conditions, either by assessing the aggregation phenotype of the strain or by plating out transconjugants. This table also contains strains in which pXO16 was naturally found and that were used as donors in mating experiments. When appropriate, the aggregation phenotype/clumping status of the strain is mentioned. Original data can be found in the followings: Reddy et al. (1987); Andrup et al. (1993); Jensen et al. (1995, 1996); Makart et al. (2018); Hinnekens et al. (2019, 2021); Hinnekens and Mahillon (2022).

Strains	Aggregation phenotype	Clumping group	Mating status	Mating condition for transfer to R strains	
				Liquid	Solid
*B. thuringiensis* sv. *israelensis* 4Q2	Agr ^+^	I	D	–	–
*B. thuringiensis* sv. *israelensis* 4Q7	Agr^−^	II	R	✓	ND
*B. thuringiensis* sv. *israelensis* 4Q7(pXO16)	Agr ^+^	I	D	–	–
*B. thuringiensis* sv. *israelensis* GBJ001/2^*^	Agr^−^	II	R	✓	✓
*B. thuringiensis* sv. *israelensis* GBJ001/2(pXO16)	Agr ^+^	I	D	–	–
*B. thuringiensis* sv. *israelensis* GSX002	Agr^−^	II	R	✓	✓
*B. thuringiensis* sv. *israelensis* BIS UM1	ND	–	D	–	–
*B. thuringiensis* sv. *israelensis* AND508	Agr ^+^	I	D	–	–
*B. thuringiensis* sv. *israelensis* NB31	Agr ^+^	I	D	–	–
*B. thuringiensis* sv. *aizawai* HD11	0	–	R	<LOD	✓
*B. thuringiensis* sv. *alesti* HD4	0	–	R	✓	✓
*B. thuringiensis* sv. *canadensis* HD224	Agr^−^	II	R	✓	ND
*B. thuringiensis* sv. *entomocidus* HD9	0	–	R	✓	✓
*B. thuringiensis* sv. *konkukian* Bt9727	ND	–	R	✓	✓
*B. thuringiensis* sv. *kurstaki* HD1	Agr^−^	II	R	✓	ND
*B. thuringiensis* sv. *kurstaki* HD73	Agr^−^	II	R	✓	ND
*B. thuringiensis* sv. *kysushensis* HD541	Agr^−^	II	R	✓	ND
*B. thuringiensis* sv. *morrisoni* HD12	Agr^−^	II	R	✓	ND
*B. thuringiensis* sv. *pakistani* HD395	Agr^−^	II	R	✓	ND
*B. thuringiensis* sv. *sotto* HD6	0	–	R	✓	✓
*B. thuringiensis* sv. *tenebrionis* BI256-82	Agr^−^	II	R	✓	ND
*B. thuringiensis* sv. *thompsoni* HD542	Agr^−^	II	R	✓	ND
*B. thuringiensis* sv. *thuringiensis* HD2	Agr^−^	II	R	✓	ND
*B. thuringiensis* sv. *thuringiensis* Bt407	ND	–	R	✓	✓
*B. thuringiensis* sv. *tolworthi* HD125	0	–	R	✓	✓
*B. thuringiensis* sv. *toumanoffi* HD201	Agr^−^	II	R	✓	ND
*B. thuringiensis* sv. *wuhanensis* HD525	Agr^−^	II	R	✓	ND
*B. cereus* 569 UM20-1	ND	–	R	✓	ND
*B. cereus* ATCC10987	Agr^−^	II	R	✓	ND
*B. cereus* ATCC10876	Agr^−^	II	R	✓	ND
*B. cereus* ATCC14579	Agr^−^	II	R	✓	ND
*B. cereus* H3081-97	Agr^−^/0	II	R	✓	✓
*B. cereus* IS075	0	–	R	✓	✓
*B. cereus* VD021	0	–	R	<LOD	✓
*B. cereus* CTMA-1571	ND	–	R	✓	✓
*B. anthracis* Weybridge UM44-1	ND	–	R	✓	ND
*B. weihenstephanensis* WSBC10204	0	–	R	✓	✓
*B. weihenstephanensis* KBAB4	0	–	R	✓	✓
*B. cytotoxicus* E28-3	0	–	R	< LOD	✓
*B. pseudomycoides* B-617	ND	–	R	–	✓
*B. mycoides* JAS391	ND	–	R	–	✓
*B. mycoides* JAS481	ND	–	R	–	✓
*B. mycoides* BPN09/1	ND	–	R	–	✓
*B. mycoides* LMG7128	ND	–	R	–	✓

* *B. thuringiensis* sv. *israelensis* GBJ001 and GBJ002 are isogenic strains, only differing in their respective antibiotic-resistance genes Str^R^ and Nal^R^.

Aggregation phenotype: Agr ^+^/clumping group I: strains containing pXO16 and acting as donors in mixed mating with Agr^−^/clumping group II strains, as described in Jensen et al., 1996 and Wiwat et al., 1995; Agr^−^/clumping group II: strains lacking pXO16 and forming aggregates in mixed mating with Agr ^+^/clumping group I strains, as described in Jensen et al., 1996 and Wiwat et al., 1995; 0: strains lacking pXO16 and forming no aggregate in mixed mating with Agr ^+^/clumping group I strains. Mating status: D, donor strain; R, recipient strain. ✓= transfer successfully demonstrated; < *LOD,* < limit of detection (~10^−7^ T/D); ND, not determined; –, not relevant.

The highly efficient mobilization capacities of pXO16 could be of concern in terms of gene circulation, including antibiotic resistance genes, if these characteristics were to express in natural settings. However, pXO16 transferability within the *B. cereus* group was only detected under tightly controlled laboratory conditions, very distant from environmental conditions. For instance, the most efficient transfer set up tested to date consists in the highly artificial filter mating technique (Hinnekens et al., 2019). Also, pXO16 exerts a significant impact on its *B. cereus s.l.* hosts, apart from its natural host, suggesting a co-adaptation between pXO16 and its “*israelensis*” host (Hinnekens and Mahillon, 2022). Taken with the natural distribution of pXO16 apparently restricted to *B. thuringiensis* sv. *israelensis* (Gillis et al., 2018), the potential impact of pXO16-mediated conjugation within the *B. cereus s.l.* in natural environments seems rather restricted. Furthermore, to the best of our knowledge, pXO16 has never been transferred by conjugation outside of the *B. cereus* group, even under solid mating conditions, suggesting a limited impact of its incredible transfer features at a wider scale.

### *Ori44*-type replicon related plasmids

The conjugative potential of the aforementioned pHT73 was first reported in early work on conjugation-like transfer within the *B. cereus* group (González et al., 1982; Wilcks et al., 1998). This *cry* plasmid encodes a *cry1Ac* gene and was successfully transferred to different members of the *B. cereus* group, including *B. mycoides*, under laboratory conditions (Hu et al., 2004). Despite successful transfer, pHT73 stability varied significantly between different recipient strains (Hu et al., 2004). Under environmental selective pressure, such instability could strongly impair the maintenance of the plasmid in those strains. Consequently, the highest stability rate was observed in “*kurstaki*” strains, indicating a biological adaptation of the plasmid to its original serotype (Hu et al., 2004). Such indications confirmed the positive correlation reported previously between the *cry* gene content of a specific strain and the strain serotype (Gaviria Rivera and Priest, 2003). pHT73 host spectrum was described as rather narrow at the time, reminiscent of the host limitations observed in early studies about pXO16 (Jensen et al., 1996). Transfer of pHT73 was later established to strains of *B. anthracis* (Yuan et al., 2010), and the compatibility of this *cry* plasmid with the anthrax plasmids pXO1 and pXO2 and the production of the Cry toxin by the “*anthracis*” recipient were also demonstrated. Such manipulations resulted in a laboratory strain of *B. anthracis* producing Cry toxin, similar to the strains of *B. cereus s.s.* and *B. anthracis* that could produce pXO12-borne Cry toxin upon conjugation-mediated acquisition of pXO12 (Battisti et al., 1985). Although the possibility of formation of such strains with hybrid phenotypes raised concerns about the spreading of insecticidal strains in the environment (Yuan et al., 2010), their actual occurrence in natural settings has so far never been reported, but remains to be further investigated.

**Figure 3:**
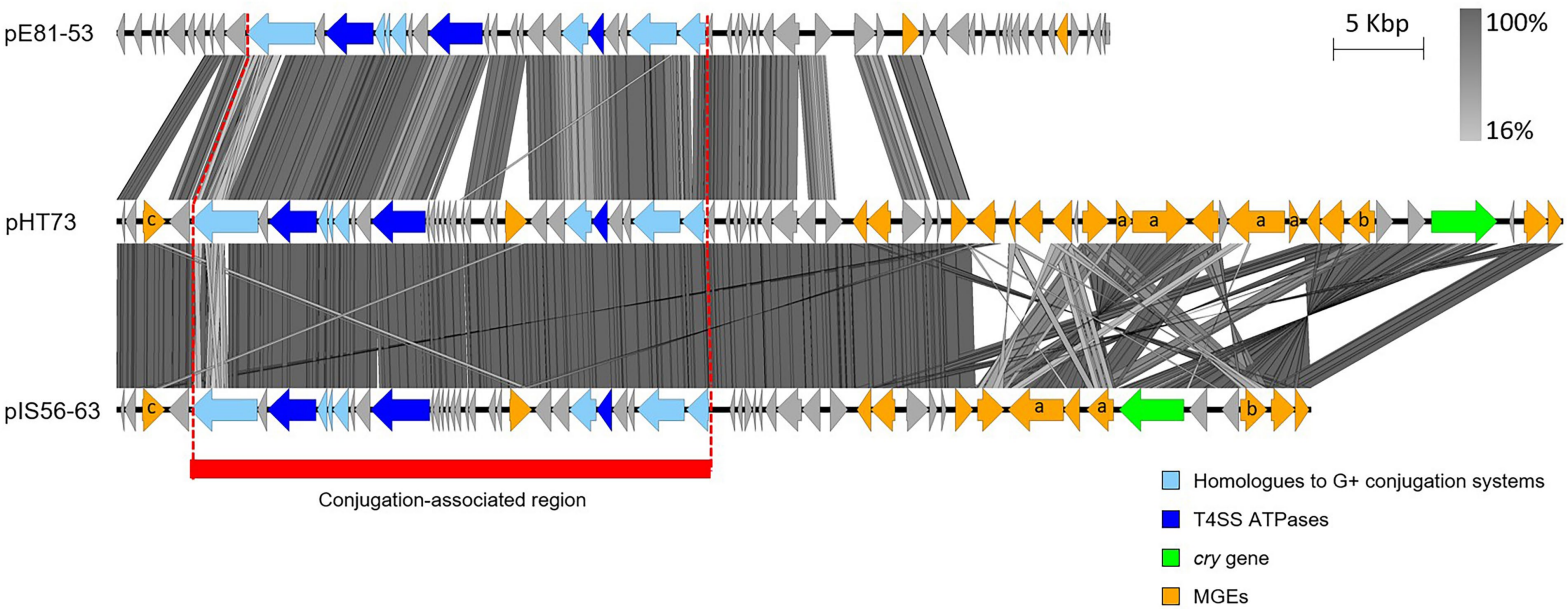
Sequence comparison of the (putative) conjugative plasmids similar to pHT73. The conjugation-associated region of the plasmids pHT73, pE81-53 and pIS56-63 share evident similarities. CDS depicted in light blue share homologies with other known conjugative systems; CDS depicted in dark blue are the putative ATPases; CDS in orange correspond to different types of MGEs (a = Tn/ b = group II intron / c = recombinase / no annotation = IS); CDS in green encode Cry toxins. The region highlighted in red corresponds to the region associated with conjugative functions. The image was generated by Easyfig 2.0 software. Genbank accession numbers: Table 1.

Other potentially conjugative plasmids share the *ori44*-type replicon of pHT73, pFR55 (Amadio et al., 2009; see Section “pAW63 and other pXO2-like plasmids”) and pIS56-63 (Murawska et al., 2014). pIS56-63 sequence exhibits strong similarities with pHT73 as it carries a *cry* gene, multiple MGEs and conjugation-related genes, even though experimental data assessing its conjugative capacities are still missing (Figure 3). Another plasmid harbors a conjugative locus related to that of pHT73, the *B. cytotoxicus* plasmid pE81-53 (Figure 3), hosted by the same strain as the pXO2-like plasmid pE81-84 (Fayad et al., 2021b). Contrary to pE81-84, experimental evidence confirmed the conjugation potential of pE81-53 (Table 1). The conjugative loci of pHT73, pE81-84 and pIS56-63 encode three putative ATPases (Figure 3) and candidates for other conjugative functions, including homologues of TcpD and TcpE, two membrane proteins essential for the conjugative system of the clostridial plasmid pCW3 (Wisniewski et al., 2015).

## *Bacillus cereus s.l.*: A pool of conjugative candidates

As stated previously, the *B. cereus s.l.* group is rich with MGEs, including those with intercellular mobility, such as plasmids, or with intracellular mobility, such as IS, class II Tn and the group-specific *B. cereus* repeats (*Bcr*). These MGEs are considered as major contributors to the genomic plasticity seen in members of this group. Many studies have attributed the ecological specificity of each member of the group to lineage-specific plasmid acquisition throughout the species’ evolution (Méric et al., 2018). These plasmids differed in number and composition. For the former, a comparison of the average number of distinct plasmids per strain among *B. cereus s.l.* species showed that *B. cereus s.s.* carries an average of 2 plasmids per cell, whereas *B. thuringiensis* average plasmid content pikes at 6–7 plasmids per cell, followed closely by *B. mycoides* at 5–6 plasmids per cell (Fayad et al., 2019). *B. thuringiensis* plasmid numbers in fact ranged between 1 to 14 distinct plasmids per cell. *B. thuringiensis* sv. *israelensis*, for example, usually harbors nine extrachromosomal molecules, among which the toxin-encoding plasmid pBtoxis and the conjugative plasmid pXO16 (Gillis et al., 2018).

While the conjugative nature of some plasmids is classically uncovered through conjugation assays, nowadays, this nature could be highlighted by the *in silico* analysis of sequenced genomes, whose numbers are increasing day-by-day. Therefore, to get a better picture at the distribution of potentially conjugative plasmids within the *B. cereus* group, we compared the sequence of plasmids from sequenced *B. cereus s.l.* genomes with the sequence of known *B. cereus s.l.* conjugative plasmids presented in previous sections. In total, 365 complete *B. cereus s.l.* genomes were recovered from the NCBI genome assembly public database (https://www.ncbi.nlm.nih.gov/assembly; last accessed June 15, 2022) mainly from the “*cereus*” (115 genomes), “*thuringiensis*” (74 genomes) and “*anthracis*” (104 genomes) species. This amounted to 1,168 plasmids (Table 3). Among the 20 plasmids reported as conjugative in the *B. cereus* group (Table 1), no sequence nor information relative to the genes involved in conjugation are available for six of them, i.e., pXO11 to pXO15 and pGB130. Six other plasmids can be grouped into four classes based on the similarity of their putative transfer apparatus: pAW63 and pBT9727 form class 1, pFR55 forms class 2, pHT73 and pE81-53 form class 3 and pBMB76 forms class 4. A class 5 comprising the case of the conjugative plasmid pXO16 will also be discussed hereafter.

**Table 3:** Identification of potential conjugative plasmids within the *B. cereus* group. Plasmids retrieved from closed genomes of *B. thuringiensis*, *B. cereus*, *B. anthracis*, *B. cytotoxicus*, *B. toyonensis*, *B. mycoides* and *B. weihenstephanensis*-*B. pseudomycoides* were analyzed for their protein sequence similarities with the model conjugative plasmids of four distinct classes. The potential conjugative plasmids encode at least two ATPase-like and one peptidoglycan hydrolase-like proteins sharing ≥60% sequence coverage and ≥35% sequence identity with the respective conjugative elements of a model conjugative plasmid.

	Number of genomes	Number of plasmids	Number of potential conjugative plasmids (%)	Number of potential conjugative plasmids per class			
				Class 1 (pAW63/pBT9727)	Class 2 (pFR55)	Class 3 (pHT73/pE81-53)	Class 4 (pBMB76)
*B. thuringiensis*	74	426	63 (14.8)	14	11	23	15
*B. cereus*	115	325	27 (8.3)	8	9	10	0
*B. anthracis*	104	151	1 (0.6)	1	0	0	0
*B. cytotoxicus*	17	32	13 (40.6)	7*	7*	6	0
*B. toyonensis*	9	26	7 (26.9)	0	5	2	0
*B. mycoides*	41	190	30 (15.8)	2	26	2	0
*B. weihenstephanensis and B. pseudomycoides*	5	18	0 (0)	0	0	0	0
Total	365	1,168	141 (12.1)	32	58	43	15

* The same seven plasmids were identified in B. cytotoxicus based on the model plasmids from class 1 and 2.

Based on proteins composition of model conjugative plasmids from each of the four classes, a tBLASTn approach was adopted to identify potential conjugative plasmids among the 1,168 analyzed *via* BLAST+ executables (Camacho et al., 2009; BLAST+ v2.12.0+). Results were then filtered based on the presence of hits with at least 60% sequence coverage and 35% sequence identity with two putative transfer ATPases and one putative conjugative peptidoglycan hydrolase. These three elements are often recognized as the basic components of conjugative T4SS (Goessweiner-Mohr et al., 2013; Schiwon et al., 2013). Plasmids previously described as conjugative (Table 1) were discarded from the results, as well as the model plasmids themselves. Overall, 12% (141 plasmids) were labelled as potentially conjugative, i.e., with self-transfer potential. Of these, 44.7% originated from *B. thuringiensis*, followed by 21.3% from *B. mycoides* and 19.1% from *B. cereus*. This was somewhat expected given that the highest number of analyzed plasmids (426, i.e., 36.47%) originated from *B. thuringiensis*.

**Figure 4:**
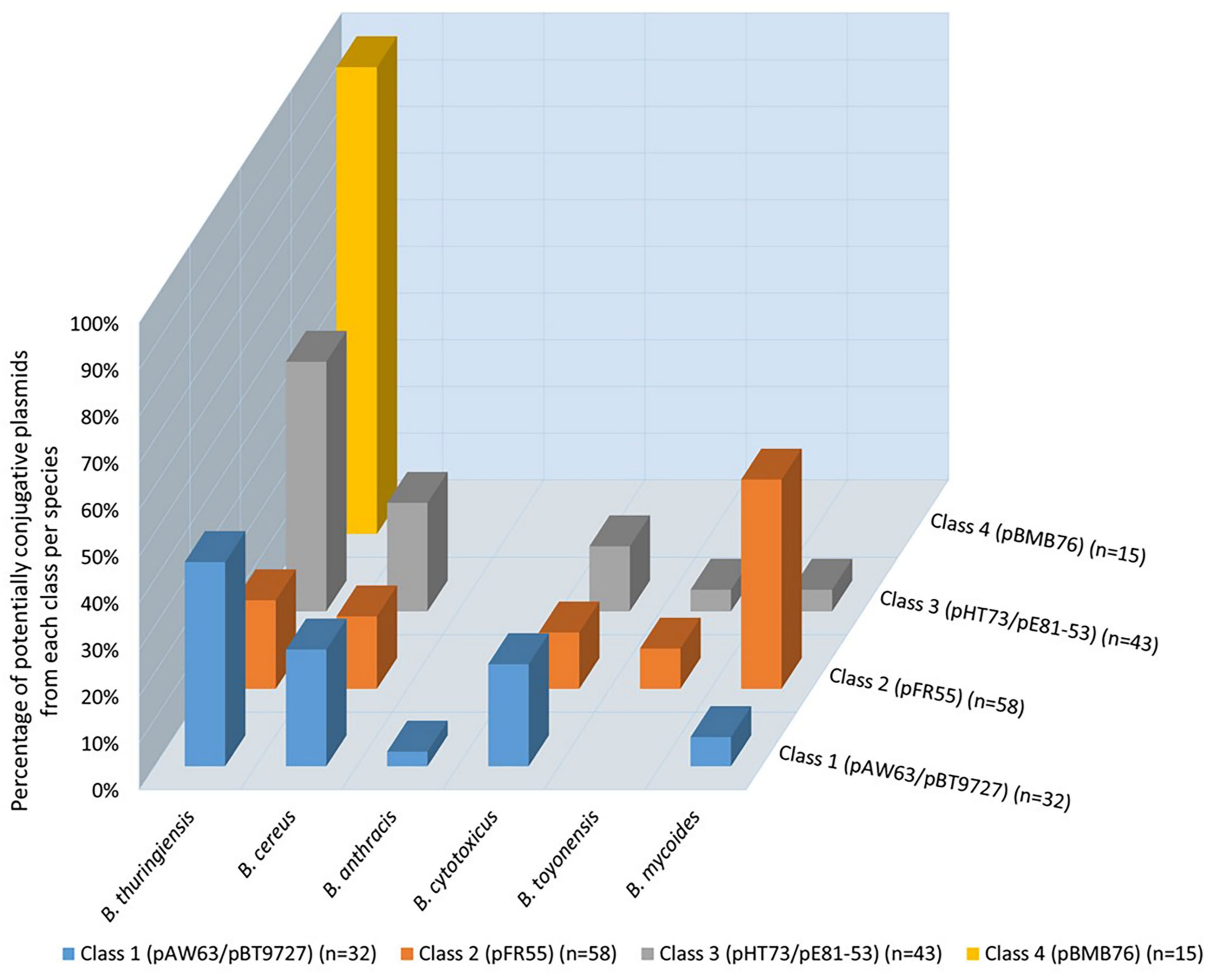
Distribution of potentially conjugative plasmids per species based on the four conjugative models (four classes). No hits were obtained with *B. weihenstephanensis* and *B. pseudomycoides*. Each bar shows the percentage of potentially conjugative plasmids identified based on the conjugative model of each of the four classes in the corresponding species. The total number of potentially conjugative plasmids identified is shown in parenthesis next to the corresponding class.

A comparison based on the four classes of conjugative plasmids adopted in this mining process showed that 23% of putative conjugative plasmids were identified with the model plasmids from class 1, 41% from class 2, 30% from class 3 and 11% from class 4. The 3D graph shown in Figure 4 highlights the distribution of the 141 putative conjugative plasmids per model per species. Although the four mined classes were of conjugative plasmids originating from *B. thuringiensis*, this was not always reflected in their distribution per species. For instance, plasmids originally from *B. mycoides* formed 44.83% of the potential conjugative pFR55-like plasmids (class 2), vs. 18.97% from *B. thuringiensis*. Class 1, i.e., pAW63/pBT9727, both originally *B. thuringiensis* plasmids, were identified mostly in *B. thuringiensis* (43.75%) with *B. cereus and B. cytotoxicus* taking on second and third places with 25 and 21.88%, respectively. The distribution of these models across species raises the questions of the early speciation inside the *B. cereus s.l.* group, and whether some of these plasmids, or their likes, date back to the early days of *B. cereus s.l*. evolution. This is one of the reasons some studies suggest that these MGEs should be ignored as to not confound the phylogenetic inference process (Bazinet, 2017).

A fifth class included the conjugative model based on the putative transfer proteins of pXO16. However, when compared to the 1,168 analyzed plasmids, nine hits from strains of *B. thuringiensis* matched, five of which are confirmed as “*israelensis*” strains. The protein sequences from these nine plasmids share at least 99.8% of identity and 99% of coverage with the corresponding protein sequences from pXO16. The reported size of those plasmids is also almost identical to the size of pXO16. The data strongly indicate that the plasmids identified in the nine *B. thuringiensis* strains are actually copies of pXO16 itself. Based on this assumption, the nine plasmids were not counted into the putative conjugative plasmids and were considered copies of the conjugative model itself.

## Conjugation into the wild and beyond

### Conjugation under “natural” environmental conditions

Few years after the first description of conjugation-like events in *B. thuringiensis*, interest for plasmid transfer under environmentally relevant conditions started with cadavers of infected lepidopteran larvae as mating matrices (Jarrett and Stephenson, 1990). Transfer was assessed by following either the mobilization of plasmids conferring a specific trait, such as *cry* plasmids (Jarrett and Stephenson, 1990) or the mobilizable Tet^R^ plasmid pBC16 (Thomas et al., 2000), or by following the transfer of a conjugative plasmid itself, like pHT73 from *B. thuringiensis* sv. *kurstaki* (Vilas-Bôas et al., 1998; Yuan et al., 2007; Santos et al., 2010) or pXO16 from *B. thuringiensis* sv. *israelensis* (Thomas et al., 2001). Over the years, transfer was evaluated in multiple infected lepidopteran larvae, including *Galleria mellonella* and *Bombyx mori* (Jarrett and Stephenson, 1990; Santos et al., 2010), in dipteran larvae (Thomas et al., 2001) and from *B. thuringiensis* donor strains to *B. thuringiensis* or *B. cereus s.s.* recipient strains. As environment-like matrices are much more complex, results were sometimes diverging. For instance, a first study stated that pHT73 could only conjugate in dead larvae among growing vegetative bacteria, after spore germination post infection (Yuan et al., 2007). However, a second study nuanced the previous results by reporting efficient conjugative transfers in environments unsuitable for the multiplication of the recipient strain (Santos et al., 2010).

Preparations of *B. thuringiensis* are widely commercialized as biopesticide to fight against various insect pests. Upon application, numerous spores are released in the environment, especially in soil, where they can survive and multiply. Subsequent studies have focused on conjugal transmission in soils to evaluate the potential dissemination of plasmid-borne genes, e.g., *cry* genes. Significant conjugative transfer of the *cry* plasmid pHT73 was reported in non-amended sterile soil, indicating that genetic exchanges between strains of *B. thuringiensis* could take place in soil (Vilas-Bôas et al., 1998, 2000). However, no transfer could be witnessed when following pBC16 mobilization in non-sterile and non-manipulated soil (Thomas et al., 2000). Instead, rapid sporulation of the strains inoculated for conjugation was observed, which most probably prevented from plasmid exchange (Thomas et al., 2000). These results validated previous observations of transfer of the conjugative plasmid pFT30 between strains of *B. cereus s.s.* and *B. subtilis* in nutrient-amended soil, while no transfer could be detected in non-amended soil (Van Elsas et al., 1987). In this early study, the *B. thuringiensis* donor strain would also quickly germinate after introduction into a soil sample.

From another study ran two decades later, only one soil-borne isolate out of 75 exhibited mobilizing activities in triparental matings, i.e., carried a conjugative plasmid (Hu et al., 2009a). Such prevalence suggested that conjugation might be rare in soil, although possible, given results described above (Hu et al., 2009a). Moreover, mating conditions used in such studies might not be representative enough of the reality, despite the evident potential for plasmid exchange. Although survival and potential conjugation in soil microcosms is essential for evaluating the impact of *B. thuringiensis*-based pesticides, the question of the impact of conjugation on the genetic structure of local populations remains. In this context, recent work surveyed the transmissibility and occurrence of conjugative plasmids in confined soil plots (Hu et al., 2020). Within two years post application, the mobility in sprayed plots was 4–5 times higher compared to control plots. Collected data suggested that application of *B. thuringiensis* in specific confined habitats may result in this population supplanting the natural *B. cereus* population and therefore boosting mobility of genetic elements associated with entomocidal properties (Hu et al., 2020). The authors however conceded that the bacterial concentrations used in their study exceeded by far the concentrations applied on field for pest management.

Application of *B. thuringiensis* sv. *israelensis*-based products to waterways is essential for the fight against dipteran larvae (e.g., mosquitoes and blackflies). Similar to work in soil microcosms, the HGT potential in river water was assessed by using the conjugative plasmid pXO16 (Thomas et al., 2001). Besides significant transfer ratio, an increase in transfer rate at the onset of parental strain sporulation was revealed, suggesting that the optimal cell growth phase for conjugation corresponds to stationary or early sporulation phases (Thomas et al., 2001). Interestingly, another environment explored for the conjugative transfer of pXO16 was the intestine of gnotobiotic rats (Wilcks et al., 2008). In parallel to many studies focusing on conjugative transfer in natural environments between strains of *B. thuringiensis*, HGT between strains of *B. anthracis* was demonstrated within a rhizosphere model set up used to decipher *B. anthracis* survival in the rhizosphere of grass plants (Saile and Koehler, 2006). Although highly important in the case of *B. cereus s.l.*, interest for transfer of genetic material in natural environments, and especially in the rhizosphere, goes beyond the *B. cereus* group (Normander et al., 1998; Sørensen and Jensen, 1998; Ronchel et al., 2000; Pearce et al., 2001).

### Conjugation in foodstuffs

For food security and health management, bacterial contamination and gene transfer in food products are key issues. Studies have revealed the potential of genetic material exchange in microenvironments, including home cooking-related situations (Kruse and Sorum, 1994), and in common food products (Cocconcelli et al., 2003). Since some members of the group are opportunistic pathogens occasionally associated with food poisoning (Dierick et al., 2005; Naranjo et al., 2011; Delbrassinne et al., 2012), *B. cereus s.l.* behavior in food products has been documented, including its DNA exchange potential. Plasmid exchange was mostly illustrated by conjugation of the *B. thuringiensis* plasmids pAW63 and pXO16, as well as by mobilization of the small plasmids pUB110 and pC194. Studies focused on liquid and semi-liquid products, including cow milk, plant-based drinks and rice pudding (Van der Auwera et al., 2007; Timmery et al., 2009; Modrie et al., 2010). Overall, conjugative transfers usually occurred at least as efficiently in food preparations as in culture broth and mobilization of small plasmids was successfully reported. Kinetics studies revealed an impact of the different food matrices on both the potential for plasmid transfer and the timing of conjugation. Compared to frequencies obtained in typical culture broth, higher transfer frequencies were obtained in food matrices, resulting from an earlier onset of conjugation, an increased transfer frequency and an extended mating time (Modrie et al., 2010). Similarly, higher transfer frequencies of pHT73 were noticed in milk compared to LB medium (Yuan et al., 2010). Gene transfer can even occur in prepacked ready-to-eat food, and not solely in private or restaurant kitchens. Indeed, pAW63 showed hasty and more efficient conjugation properties in salt conditions mimicking those commonly found in food products like cheese and sausages (Beuls et al., 2012).

In a study looking at *B. cereus* strains used in probiotics, a large proportion of isolated strains were resistant to tetracycline and one of these strains harbored the genetic determinants of tetracycline resistance on an MGE, presenting a risk of antibiotic-resistance gene transfer (Zhu et al., 2016). From other strains of *B. cereus s.l.* isolated from both raw and ultra-heat-treated milk, around 20% of the non-*B. anthracis* strains carried pXO1-like and/or pXO2-like plasmids (Bartoszewicz and Marjańska, 2017). As described previously, the family of pXO2-like plasmids are well-known for their conjugative potential (i.e., pAW63 and pBT9727, see Section “pAW63 and other pXO2-like plasmids”), emphasizing the potential for conjugation in this case study. Based on work cited hereinabove, food products are proposed to favor exchange of genetic material (Bartoszewicz and Marjańska, 2017). By providing richer media, foodstuffs might indeed sustain bacterial proliferation, resulting in higher bacterial density, extended mating periods and more efficient plasmid transfer (Bartoszewicz and Marjańska, 2017).

### Conjugation in confined environments

Bacteria are well-known for their adaptability to challenging environments and for their amazing genetic flexibility due to HGT. Strictly confined environments, such as the Antarctic Concordia Station (ACS) or the International Space Station (ISS), are especially rough due to space confinement, high levels of irradiation and/or microgravity. Microbial genetic exchange in such conditions raised scientists’ interest due to potential acquisition of new pathogenic traits. In spacecraft, modifications in microbiome could threaten astronaut health. Significant number of studies evaluated potential effects of real or simulated microgravity on bacterial characters, including growth, phenotype, virulence, metabolism and gene transfer (Senatore et al., 2018; Prasad et al., 2020; Bijlani et al., 2021; Mahnert et al., 2021). Most of these studies concluded in a strong influence of microgravity on bacterial communities, potentially leading to the evolution of space-specific properties that would not arise on Earth. Research groups also focused on the potential effects of microgravity on HGT. While an increase in genetic exchange between strains of *Acinetobacter* and *Staphylococcus* co-cultivated under simulated microgravity was reported, the root cause of this stimulation of genetic transfer was not investigated and transformation was proposed as the main underlying transfer mechanism (Urbaniak et al., 2021). Another research study examined strains of *Staphylococcus* and *Enterococcus* isolated from the ISS and the ACS for their conjugative properties. More than 80% of ISS strains and over 50% of ACS strains actually carried plasmids encoding signature transfer genes from well-characterized Gram-positive conjugative plasmids, like pIP501 (Schiwon et al., 2013).

Studying conjugation under space or confined conditions among the members of the *B. cereus* group was not left behind, particularly for strains of *B. thuringiensis*. The first study investigating plasmid-mediated conjugation under space flight conditions involved triparental matings of Gram-positive or Gram-negative bacteria and used *B. thuringiensis* as a Gram-positive model. Not only was it established that genetic exchange could occur by conjugation in space flight, but it was also suggested that conjugation and mobilization capacities were increased in the Gram-positive model (De Boever et al., 2007). However, such conclusions must be nuanced by the poor experimental controls and unexplained negative results in ground-based experiments. Furthermore, work published a couple of years later reported no statistically significant differences in conjugation and mobilization efficiency of *B. thuringiensis* in simulated microgravity – using three different simulation set-ups – compared to standard laboratory conditions (Beuls et al., 2012). Although no stimulation of genetic transfer was revealed, neither was inhibition. Further evidence of HGT in confined space habitats were gathered when one *B. cereus s.l.* strain, putatively harboring a conjugative plasmid, was identified among more than 40 *Bacillus* strains isolated from ISS and ACS and tested for conjugative abilities (Timmery et al., 2011). Strains were also tested for their capacity to receive foreign DNA by HGT, mimicking their potential recipient role in conjugative matings. Overall, two strains of *B. cereus s.l.* could acquire the conjugative plasmids pAW63 and pXO16, and four additional strains successfully acquired pXO16 (Timmery et al., 2011). Concerning pXO16 transfer, recent work demonstrated an extended host range of the plasmid *via* solid mating instead of liquid mating (Hinnekens et al., 2019). One should then consider the possibility that more ISS and ACS strains could act as pXO16 recipients when assessing transfer in solid matings. Altogether, collected data indicated that (i) most *B. cereus s.l.* strains analyzed for their plasmid profiles carried at least one plasmid and (ii) plasmid-mediated transfer, i.e., conjugation, could occur between strains of *B. cereus s.l.* originating from space environments and in space-mimicking conditions (De Boever et al., 2007; Beuls et al., 2009; Timmery et al., 2011). In light of astronaut health, such considerations are essential as modifications in “strain-plasmid” association could lead to harmless strains developing pathogenic characteristics (Timmery et al., 2011).

## Concluding remarks and outlooks

Plasmids are key players in the lifestyle of *B. cereus s.l.*, whether they encode major toxins, provide niche-specific traits, or promote exchange of genetic material. When it comes to conjugation, although a significant proportion of plasmids from *B. cereus s.l.* show potential for self-transferability, more elements come into play. Despite studies analyzing conjugal transfer in specific environments, like insect larvae, soil, foodstuff or confined environments, conjugation was mostly examined in laboratory environments, under highly artificial mating conditions. This applies to studies both in *B. cereus s.l.* and other bacterial groups. However, the natural environment for conjugation seems much more complex, likely occurring in dense bacterial communities in diverse natural niches (Shen et al., 2022). Furthermore, the different members of the *B. cereus* group tend to occupy different environmental niches, limiting their contact and interaction, and thereby limiting conjugation events. This is particularly true for *B. anthracis* which displays a specific and restricted natural lifestyle (Jensen et al., 2003). Moreover, all strains exhibiting a hybrid phenotype, e.g., strains of *B. anthracis* or *B. cereus s.s.* with *B. thuringiensis cry* plasmids, were so far obtained in laboratory settings and not isolated from natural environments (Battisti et al., 1985; Yuan et al., 2010).

In addition to the limited occurrence of “natural” conjugation *per se*, it is important to consider the relative stability of plasmids in different hosts. Broad-host spectrum plasmids have evolved to adapt to multi-host environments. However, in restricted niches, studies showed that in the hosts, plasmids have specifically adapted to alleviate their metabolic cost (Kottara et al., 2016). In other bacteria, maintaining such host-specific plasmids might be more costly, limiting the success of potential transfer overtime. Plasmid stability in a defined host relies on complex interactions between the plasmid itself, the host and the environment, which interact together to determine the balance between plasmid carriage burden and the profit from plasmid-borne genes (Hall et al., 2015; Kottara et al., 2018). More insight into the overall impact and risk of conjugation in the *B. cereus* group, and in other bacterial species, should be brought by further studies focusing on conjugation under environmental-relevant conditions, within complex bacterial communities.

## Author contributions

PH, NF, and JM: conceptualization, methodology, validation, and writing – original draft preparation. PH and NF: Software, formal analysis, investigation, and data curation. JM: resources, Supervision, project administration, and funding. PH, NF, AG, and JM: writing – review and editing. All authors read and approved the final manuscript.

## Funding

This work was supported by the National Foundation for Scientific Research (FNRS, Belgium – Research grants FNRS-CDR J.0144.20 to J.M. and FNRS 1.A682.19 to PH), by the International Office for Cooperation of the *Université catholique de Louvain* (UCLouvain; Bursaries to NF), and the Research Department of the Communauté française de Belgique (Concerted Research Action, ARC 17/22–084 grant to JM).

## Conflict of interest

The authors declare that the research was conducted in the absence of any commercial or financial relationships that could be construed as a potential conflict of interest.

## Publisher’s note

All claims expressed in this article are solely those of the authors and do not necessarily represent those of their affiliated organizations, or those of the publisher, the editors and the reviewers. Any product that may be evaluated in this article, or claim that may be made by its manufacturer, is not guaranteed or endorsed by the publisher.
